# Numerical exploration of slip effects on second-grade fluid motion over a porous revolving disk with heat and mass transfer

**DOI:** 10.1016/j.heliyon.2023.e18683

**Published:** 2023-07-26

**Authors:** Haleema Sadia, M. Mustafa

**Affiliations:** School of Natural Sciences (SNS), National University of Sciences and Technology (NUST), Islamabad, 44000, Pakistan

**Keywords:** Revolving disk, Similarity solution, Elasticity parameter, Partial slip, Heat transfer, Computational result

## Abstract

Revolving-disk systems are employed in various industrial settings including turbine engineering, chemical and food processing industries and others. The current article scrutinizes a second-grade fluid motion generated by an infinite porous disk having partial slip character. Heat transfer induced by heating of the disk surface and by viscous and ohmic heating effects is modeled and analyzed under thermal slip condition. Accompanying mass transfer process with thermophoretic diffusion is also formulated. A self-similar system is obtained akin to the case of no-slip case discussed in a previously published study. The adoption of velocity slip assumption induces non-linearity in the boundary conditions in velocity components. Computational procedure embedded in MATLAB bvp4c platform is opted to simulate the system for full range of slip parameters. In contrast to a previously published work pertaining to the no-slip case, present numerical methodology gives accurate results for wide ranges of Prandtl number and elasticity parameter. Boundary layer formations above the disk are examined under various controlling parameters. A comparative assessment of slip and no-slip cases is presented through both graphical illustrations and tabulated results for the resisting torque, the Nusselt number and the Sherwood number. Current numerical findings match very well with the existing homotopy solutions for the no-slip case. The presence of a wall slip mechanism leads to a clear suppression of all the velocity components. Furthermore, an augmentation in the thermal/concentration slip coefficient significantly reduces the thermal/solutal penetration depth. Additionally, we observe a noticeable increase in the driving torque as the elasticity parameter enhances. The slip action of the surface is also predicted to raise the torque required by the disk.

## 1 Introduction

It is well known that fluids involved in industries, especially in the chemical and related processing industries, do not obey Newton's relationship between stress and strain rate. Examples include manufactured coatings, eatables (such as cheese, melted chocolate, gelatin, mayonnaise, whipped cream and yoghurt), slurries (such as emulsions, cement slurry, paper pulp and foams), multi-state mixtures (such as liquid-gas diffusion, oil-water ointment etc.) and biological fluids (blood, mucus etc.). Mathematical and computational analysis with non-Newtonian fluids offer challenging problems to the non-linear differential equations' experts. Moreover, heat transport mechanism in non-Newtonian liquids has been the focus of research interest for many years, and tremendous research contributions to this field have illuminated diverse physical aspects of the subject matter. Non-Newtonian flow behavior is manifested in a variety of ways and no single stress-strain relation adequately summarizes all the characteristics of non-Newtonian flows. For instance, the widely recognized generalized Newtonian models (such as power-law model [1], Carreau model [2] etc.) cover the shear thinning/thickening response but these are unable to depict elastic effects. Another class includes viscoelastic substances which are formed by a viscous component and an elastic one. Usually, the viscoelastic fluids are combination of solvents and polymers. For such fluids, shear stress is a memory function of the strain rate. That is, when the applied shear force is removed, the shear stress gradually decreases; a phenomenon known as stress relaxation.

Different viscoelastic fluid models have been developed based on their rheological features. Perhaps, the second-grade viscoelastic model suggested by Rivlin and Ericksen [3] is the most widely studied model in literature. This model emphasizes normal stress differences that are present in various fluids (polymeric solutions, liquid crystals, suspensions etc.). The viscosity coefficient appearing in second-grade fluid model is assumed constant, which is not an ideal assumption for fluids with high viscosities that rely on shear rate. Pioneering works of Kaloni [4], Rajagopal and Gupta [5], Pontrelli [6] and a few others have led to many subsequent research initiatives on boundary layer formations in viscoelastic flows. Literature is full of such studies where second-grade model has been adopted to investigate different flow situations which include flow past a cavity [7], peristaltic flows [[8], [9], [10]] etc., nanoparticle working fluid assumptions [11,12] etc., mixed convection flows [13,14] etc., channel flow [15], Soret and Dufour effects [16,17] to mention a few.

Rotating-disk systems are widely seen in several industrial setups including turbine engineering, food processing technologies, computer disk drivers, evaporators etc. Commonly discussed are the situations of rotating disk in an otherwise stationary environment and stationary disk in a revolving fluid at a constant angular velocity. Former case, which is the subject of this study, was first recognized by Von-Karman [18], who analyzed the problem using an approximate momentum integral scheme. Different physical effects have been incorporated to scrutinize the Von-Karman rotating disk model which include permeable wall [19], vertically moving disk assumption [20], nanofluid assumption [[21], [22], [23]] etc. and shrinking disk effects [24,25] etc. In addition to the above-stated studies, information about non-Newtonian effects in the classical Von-Karman's configuration is also available. For example, Elliot [26] introduced a modeling framework for revolving-disk triggered viscoelastic flow using the Walters-B model. A perturbation solution was computed via Runge-Kutta integration scheme. Effects of elasticity on the induced viscoelastic flow were enlightened with the aid of both graphical illustrations and numerical data. Results of ref. [26] reveals that the impact of elasticity is to amplify the magnitude of the velocity components and reduce the magnitude of the turning moment exerted on the disk. Later, Ariel [27] conducted a comprehensive computational study on the boundary layer formation above a revolving disk in an otherwise static second-grade fluid. In Ref. [27], a robust finite difference procedure was developed for producing numerical results which were found to be in complete compliance with the obtained perturbation and asymptotic approximations. Andersson et al. [28] focused on the numerical solutions for rotating-disk system involving power-law fluids and successfully generated accurate findings for both pseudo-plastic and dilatant type shear-thinning fluids. The findings indicate that the impact of the magnetic field is more noticeable in shear-thinning fluids compared to shear-thickening fluids. Local similarity analysis for Von-Karman situation involving Bingham fluid was put forward by Ahmadpour et al. [29]. The study shows that an increase in the yield stress is predicted to result in an increase in the tangential velocity. Furthermore, the work of ref. [29] was revisited by Guha and Sengupta [30] through different approaches. Streamlines, isotherms and velocity contours were generated from the simulations. Reiner-Rivlin model has also been adopted by different authors (e.g. Sahoo et al. [31], Tabassum and Mustafa [32], Rashid and Mustafa [33], Rafiq et al. [34] etc.) to examine heat transfer mechanism in rotating plane induced non-Newtonian motion.

Quite recently, viscous dissipation effects on the Von-Karman flow problem were formulated using Walters-B and second grade models by Burhan and Mustafa [35,36] respectively, when slip boundary effects were missing. Current study extends the work [36] by including partial slip boundary assumption for both velocity and temperature functions and by considering concentration distribution. The current study is the first to investigate the slip flow of a second-grade fluid over a rotating disk, as no prior reports have addressed this particular topic. The study of coupled heat and mass transfer in a viscoelastic fluid motion across a permeable revolving disk under slip conditions is motivated by the need to understand the behavior of viscoelastic fluids at the disk surface-fluid interfaces. The relative motion or slip between the fluid and the solid surface is referred to as slip conditions. In this context, investigating slip conditions is critical since they can considerably impact the system's heat and mass transport characteristics. Slip situations are prevalent in real-world systems involving viscoelastic fluids, such as polymer solutions or suspensions. Understanding the impacts of partial slip effect on heat and mass transfer is important for accurately optimizing the heat transfer rates of many processes in chemical engineering, such as polymer processing, mixing and reactive flows. Partial slip assumptions also produce a non-linear Robbin-type boundary condition in velocity components. Like refs. [35,36], quadratic surface temperature/concentration assumption is invoked to ensure an exact similarity analysis of the model. Different from Ref. [36], a collocation method embedded in bvp4c routine of MATLAB is applied to produce self-similar results for full range of slip parameters. Other commonly employed methods for solving boundary value problems include Runge-Kutta method combined with shooting technique (see Refs. [37,38]) etc. Here the bvp4c based collocation approach is preferred because of its higher accuracy (having fourth-order convergence), robustness and a user-friendly interface. Contribution of second-grade fluid assumption on the slip flow with heat/mass transfer is studied through detailed plots of non-dimensional velocities, temperature and concentration. Furthermore, values of moment coefficient, Nusselt number and Sherwood number are tabulated and compared with the corresponding homotopy results in the no-slip case.

## 2 Problem formulation

**Fig. 1:**
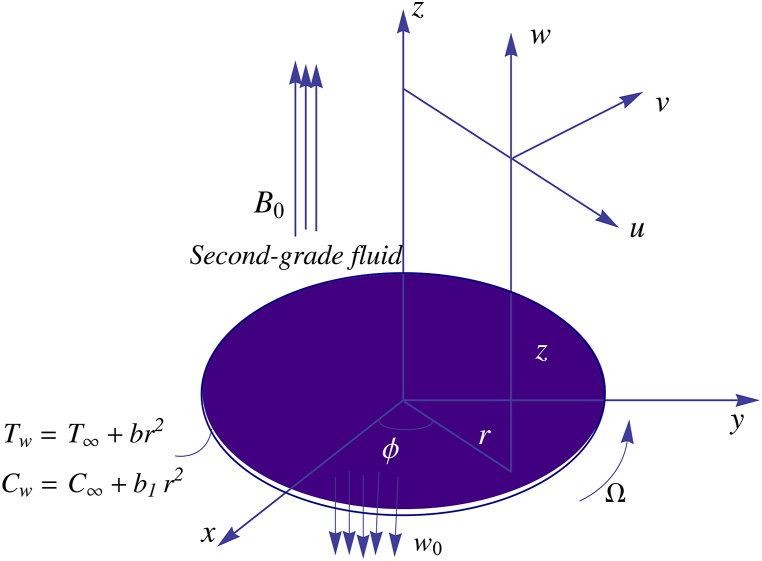
A schematic for flow over a porous rotating disk.

Suppose a viscoelastic fluid (such as slurries, polymeric liquids, biological fluids etc.) flows over a revolving heated disk residing in the xy-plane. The disk, supposed to be rough and permeable, admits rotations about the vertical axis (r=0) with rotation rate Ω. Both heat and mass transfer mechanisms are studied here. A widely preferred second-grade model based on Rivlin-Eriksen tensor is adopted. We adopt a cylindrical coordinate system (r,φ,z) with corresponding velocities being denoted by (u,v,w) respectively (see Fig. 1). An electrically conducting viscoelastic fluid is exposed to a transverse magnetic field having strength $B_{0}$, with the usual assumption of small magnetic Reynolds number. Present paper accounts for the following new assumptions:

o Partial slip conditions are adopted, that will generate non-linearity in the boundary conditions in radial and axial velocities.
o Surface temperature $T_{w}$ and concentration at the surface $C_{w}$ are supposed to change in the radial direction as $T_{w}=T_{\infty }+br^{2}$ and $C_{w}=C_{\infty }+b_{1}r^{2}$ where b and $b_{1}$ are constants while $T_{\infty }$ and $C_{\infty }$ represent the ambient temperature and concentration respectively.
o Both viscous dissipation and Joule heating mechanisms are accounted for.
o Mass transfer process is influenced by thermophoretic diffusion.

Invoking the above assumptions and the boundary layer approximations, relevant equations are presented below [36]:

(1) $$\frac{\partial }{\partial r}\left(ru\right)+\frac{\partial }{\partial z}\left(rw\right)=0$$

(2) $$u\frac{\partial u}{\partial r}+w\frac{\partial u}{\partial z}-\frac{v^{2}}{r}=\nu \frac{\partial^{2}u}{\partial z^{2}}+\frac{\alpha_{1}}{\rho }\left\{\begin{array}{c} u\frac{\partial^{3}u}{\partial r\partial z^{2}}+w\frac{\partial^{3}u}{\partial z^{3}}+\frac{\partial u}{\partial r}\frac{\partial^{2}u}{\partial z^{2}}-\frac{\partial u}{\partial z}\frac{\partial^{2}w}{\partial z^{2}}\\ +\frac{\partial^{2}v}{\partial z^{2}}\left(\frac{\partial v}{\partial r}-\frac{v}{r}\right)+\frac{\partial v}{\partial z}\frac{\partial^{2}v}{\partial r\partial z}-\frac{1}{r}{\left(\frac{\partial u}{\partial z}\right)}^{2} \end{array}\right\}-\frac{\sigma B_{0}^{2}}{\rho }u$$

(3) $$u\frac{\partial v}{\partial r}+w\frac{\partial v}{\partial z}+\frac{uv}{r}=\nu \frac{\partial^{2}v}{\partial z^{2}}+\frac{\alpha_{1}}{\rho }\left\{\begin{array}{c} -\frac{\partial v}{\partial z}\frac{\partial^{2}u}{\partial r\partial z}+u\frac{\partial^{3}v}{\partial r\partial z^{2}}+w\frac{\partial^{3}v}{\partial z^{3}}+\frac{u}{r}\frac{\partial^{2}v}{\partial z^{2}}\\ -\frac{\partial v}{\partial z}\frac{\partial^{2}w}{\partial z^{2}}-\frac{1}{r}\frac{\partial u}{\partial z}\frac{\partial v}{\partial z} \end{array}\right\}-\frac{\sigma B_{0}^{2}}{\rho }v$$

(4) $$u\frac{\partial T}{\partial r}+w\frac{\partial T}{\partial z}=\frac{k}{\rho C_{p}}\frac{\partial^{2}T}{\partial z^{2}}+\sigma B_{0}^{2}\left(u^{2}+v^{2}\right)+\frac{\mu }{\rho C_{p}}\left\{{\left(\frac{\partial u}{\partial z}\right)}^{2}+{\left(\frac{\partial v}{\partial z}\right)}^{2}\right\}+\frac{\alpha_{1}}{\rho C_{p}}\left(u\frac{\partial u}{\partial z}\frac{\partial^{2}u}{\partial r\partial z}+u\frac{\partial v}{\partial z}\frac{\partial^{2}v}{\partial r\partial z}+w\frac{\partial u}{\partial z}\frac{\partial^{2}u}{\partial z^{2}}+w\frac{\partial v}{\partial z}\frac{\partial^{2}v}{\partial z^{2}}\right)$$

(5) $$u\frac{\partial C}{\partial r}+w\frac{\partial C}{\partial z}=D_{B}\frac{\partial^{2}C}{\partial z^{2}}+\frac{D_{T}}{T_{\infty }}\frac{\partial^{2}T}{\partial z^{2}}$$

In Eqs. (1), (2), (3), (4), (5), T gives the local temperature, p shows pressure function, ρ is constant fluid density, $C_{p}$ gives the specific heat capacity, k expresses the constant thermal conductivity, C shows the concentration field, $D_{B}$ stands for mass diffusivity coefficient and $D_{T}$ stands for the thermophoretic diffusion coefficient.

Above equations are to be solved subject to the following constraints:

(6) $$u=\lambda_{1}\tau_{rz},v=\lambda_{1}\tau_{\theta z}+r\omega ,w=-w_{0},T=T_{w}+\lambda_{2}\frac{\partial T}{\partial z},C=C_{w}+\lambda_{3}\frac{\partial C}{\partial z}\;at\;z=0\text{,}$$

$$u\to 0,\frac{\partial u}{\partial z}\to 0,v\to 0,\frac{\partial v}{\partial z}\to 0,T\to T_{\infty },C\to C_{\infty }\;as\;z\to \infty$$

where $\tau_{rz}$ and $\tau_{\theta z}$ show radial and azimuthal stresses, $\lambda_{1},\lambda_{2}$ and $\lambda_{3}$ are the coefficients of velocity, thermal and concentration slips respectively and $w_{0}$ characterizes wall transpiration effect at the disk surface. For the provision of self-similar numerical solutions, we proceed with the transformations:

(7) $$u=r\Omega f^{\prime }\left(\xi \right),v=r\Omega g\left(\xi \right),w=-2\sqrt{\nu \Omega }f\left(\xi \right),\theta \left(\xi \right)=\frac{T-T_{\infty }}{T_{w}-T_{\infty }},\varphi \left(\xi \right)=\frac{C-C_{\infty }}{C_{w}-C_{\infty }}\text{,}$$

with $\xi =z{\left(\Omega /\nu \right)}^{\frac{1}{2}}$ denoting the similarity variable. It can be shown that transformations (7), produce the following set of differential equations:

(8) $$f^{\prime \prime \prime }+g^{2}+2ff^{\prime \prime }-f^{\prime 2}+K\left(2f^{\prime }f^{\prime \prime \prime }+f^{\prime \prime 2}-g^{\prime 2}-2ff^{4}\right)-Mf^{\prime }=0\text{,}$$

(9) $$g^{\prime \prime }-2f^{\prime }g+2fg^{\prime }+K\left(-2fg^{\prime \prime \prime }+2f^{\prime }g^{\prime \prime }\right)-Mg=0$$

(10) $$\theta^{\prime \prime }+2\;\mathrm{Pr}\left(f\theta^{\prime }{-f}^{\prime }\theta \right)+PrEc\left(f^{\prime \prime 2}+g^{\prime 2}\right)+MEcPr\;\left(f^{\prime 2}+g^{2}\right)+KPrEc\left(f^{\prime }f^{\prime \prime 2}+f^{\prime }g^{\prime 2}-2ff^{\prime \prime }f^{\prime \prime \prime }-2fg^{\prime }g^{\prime \prime }\right)=0$$

(11) $$\varphi^{\prime \prime }+2Sc\left(f\varphi^{\prime }{-f}^{\prime }\varphi \right)+ScSr\theta^{\prime \prime }=0$$

The boundary conditions (6) are transformed as follows:

(12a) $${f\left(0\right)=A,f}^{\prime }\left(0\right)-\beta f^{\prime \prime }\left(0\right)-2K\beta \left\{f^{\prime }\left(0\right)f^{\prime \prime }\left(0\right)-Af^{\prime \prime \prime }\left(0\right)\right\}=0,g\left(0\right)-\beta g^{\prime }\left(0\right)-2K\beta \left\{f^{\prime }\left(0\right)g^{\prime }\left(0\right)-Ag^{\prime \prime }\left(0\right)\right\}-1=0,\theta \left(0\right)-\gamma \theta^{\prime }\left(0\right)-1=0,\varphi \left(0\right)-\gamma_{1}\;\varphi^{\prime }\left(0\right)-1=0$$

(12b) $$f^{\prime }\to 0,f^{\prime \prime }\to 0,g\to 0,g^{\prime }\to 0,\theta \to 0,\varphi \to 0\;as\;\zeta \to \infty$$

In Eqs. (8), (9), (10), (11), (12a), (12b), K is referred as the elasticity parameter, A is termed as permeability parameter, M is mentioned as magnetic interaction parameter, Pr defines Prandtl number, Ec expresses Eckert number, Sr shows thermophoresis parameter, Sc gives Schmidt number and β,γ and $\gamma_{1}$ are stated as velocity slip, thermal slip and concentration slip parameters respectively. Mathematical expressions of these parameters are presented as follows:

(13) $$K=\frac{\alpha_{1}\Omega }{\mu },A=\frac{w_{0}}{2\sqrt{\nu \Omega }},M=\frac{\sigma B_{0}^{2}}{\rho \Omega },\beta =\lambda_{1}\mu {\left(\frac{\Omega }{\nu }\right)}^{1/2},\gamma =\lambda_{2}{\left(\frac{\Omega }{\nu }\right)}^{1/2}$$

$$\gamma_{1}=\lambda_{3}{\left(\frac{\Omega }{\nu }\right)}^{1/2},\mathrm{Pr}=\frac{\mu c_{p}}{k},Ec=\frac{\Omega^{2}}{bc_{p}},Sc=\frac{\nu }{D_{B}},S_{r}=\frac{D_{T}b}{T_{\infty }b_{1}\nu }$$

To estimate the torque required in revolving the disk of radius R, we solve the following definite integral:

(14) $$T_{r}=\int_{0}^{r}\tau_{\theta z}{|}_{z=0}\;2\pi r^{2}\;dr$$

Furthermore, defining the moment coefficient as $C_{m,r}=T_{r}/\rho \Omega^{2}r^{5}$ and using transformations (7), one can prove the following result:

(15) $${2\sqrt{{Re}_{r}}C}_{m,r}=\pi \left[g^{\prime }\left(0\right)+K\left\{4f'\left(0\right)g'\left(0\right)-2f\left(0\right)g^{\prime \prime }\left(0\right)\right\}\right]$$

in which ${Re}_{r}=r^{2}\Omega /\nu$ defines the local Reynolds number.

Other subtle relations include the Nusselt number Nu, Sherwood number Sh and volume flow rate Q through disk of radius R which are obtained as follows: $Nu=-\theta^{\prime }\left(0\right)$, $Sh=-\varphi^{\prime }\left(0\right)$ and $Q=2\sqrt{\nu \Omega }f\left(\infty \right)\pi R^{2}$.

## 3 Procedure for numerical solutions

Eqs. (8), (9), (10), (11) along with conditions (12a) and (12b) constitute a coupled non-linear system which has no exact solution. For numerical evaluation of the fourth-order system, an expedient MATLAB package bvp4c is applied. This package applies a collocation method that approximates the solution with a C1-continuous polynomial, which makes it useful for tackling a variety of boundary value problems. Also, collocation method has accuracy of fourth-order meaning that the numerical solution converges at a rate proportional to fourth power of the mesh size. Such high accuracy is crucial for obtaining reliable numerical results in different applications. In order to use the package, the equivalent first-order equations need to be computed. In this regard, we make the following substitutions:

$$Y_{1}=f,Y_{2}=f^{\prime },Y_{3}=f^{\prime \prime },Y_{4}=f^{\prime \prime \prime },Y_{5}=g,Y_{6}=g^{\prime },Y_{7}=g^{\prime \prime },Y_{8}=\theta ,Y_{9}=\theta^{\prime },Y_{10}=\varphi ,Y_{11}=\varphi^{\prime }$$

First-order system is obtained as follows:

(16) $$Y_{4}^{\prime }=\frac{Y_{4}+{Y_{5}}^{2}+2Y_{1}Y_{3}-{Y_{2}}^{2}+K\left(2Y_{2}Y_{4}+{Y_{3}}^{2}+{Y_{6}}^{2}\right)-MY_{2}}{2KY_{1}}$$

(17) $$Y_{7}^{\prime }=\frac{Y_{7}+2{KY}_{2}Y_{7}-2Y_{2}Y_{5}+2Y_{1}Y_{6}-MY_{5}}{2KY_{1}}$$

(18) $${Y_{9}}^{\prime }=\mathrm{Pr}\left(2Y_{2}Y_{8}-2Y_{1}Y_{9}\right)-EcPr\left({Y_{3}}^{2}+{Y_{6}}^{2}\right)-KEcPr\left({{Y_{2}Y}_{3}}^{2}+{{Y_{2}Y}_{6}}^{2}-2Y_{1}Y_{3}Y_{4}-2Y_{1}Y_{6}Y_{7}\right)-MEcPr\left({Y_{2}}^{2}+{Y_{5}}^{2}\right)$$

(19) $$Y_{11}^{\prime }=Sc\left(2Y_{2}Y_{10}-2Y_{1}Y_{11}\right)-ScSrY_{9}^{\prime }$$

Eqs. (16), (17), (18), (19) are inserted in MATLAB bvp4c code along with mesh size and the initial guesses. For detailed procedure, reader is referred to the article [29].

## 4 Numerical results and discussion

In this section, an insightful analysis on the physical effects of controlling parameters (defined in Eq. (13)) on the velocity (all components) and temperature is conducted. Before moving on to discuss the physical interpretation of the results, it is important to perform a validation study. Table 1 demonstrates an excellent correlation between the current results and the computational findings provided by Ariel [27] and Burhan and Mustafa [36]. Results of resisting torque (shown in Eqs. (14), (15)) are compared with the homotopy results computed in Ref. [36] (see Table 2) in the no slip case, and such comparison is excellent.

**Table 1:** A comparison of present numerical results with those obtained by Ariel [27] and Burhan and Mustafa [36] for different values of M when K=A=0.

M	Ariel [27]		Burhan and Mustafa [37]		Present	
	$f^{\prime \prime }\left(0\right)$	${-g}^{\prime }\left(0\right)$	$f^{\prime \prime }\left(0\right)$	${-g}^{\prime }\left(0\right)$	$f^{\prime \prime }\left(0\right)$	${-g}^{\prime }\left(0\right)$
0.2	0.453141	0.708795	0.453129	0.708793	0.453122	0.708725
0.4	0.405576	0.802376	0.405576	0.802376	0.405576	0.802375
0.6	0.366698	0.894476	0.366698	0.894476	0.366698	0.894476
0.8	0.335092	0.983607	0.335090	0.983607	0.335090	0.983607
1.0	0.309258	1.069053	0.309258	1.069053	0.309258	1.069053
1.2	0.287915	1.150635	0.287915	1.150635	0.287915	1.150634
1.4	0.270049	1.228466	0.270049	1.228466	0.270049	1.228466
1.6	0.254892	1.302793	0.254892	1.302793	0.254893	1.302793
2.0	0.230559	1.442094	0.230559	1.442094	0.230560	1.442093

**Table 2:** A comparison of present numerical results of resisting torque (given by Eq. (15)) with those obtained by Burhan and Mustafa [36] for different values of K,A and M.

K	A	M	$\frac{2}{\pi }{Re_{r}}^{\frac{1}{2}}C_{m,r}$	
			HAM results [36]	Present Numerical scheme
0	0.5	1	−1.65707	−1.65707
0.25			−1.79271	−1.78751
0.5			−1.87536	−1.87536
1			−2.00000	−2.00000
0.5	0.15		−1.27874	−1.27359
	0.3		−1.52255	−1.52255
	0.5		−1.87536	−1.87536
	0.5	0	−1.17555	−1.17537
		0.4	−1.48226	−1.48226
		1	−1.87536	−1.87536
		2.5	−2.64959	−2.64960

**Fig. 2:**
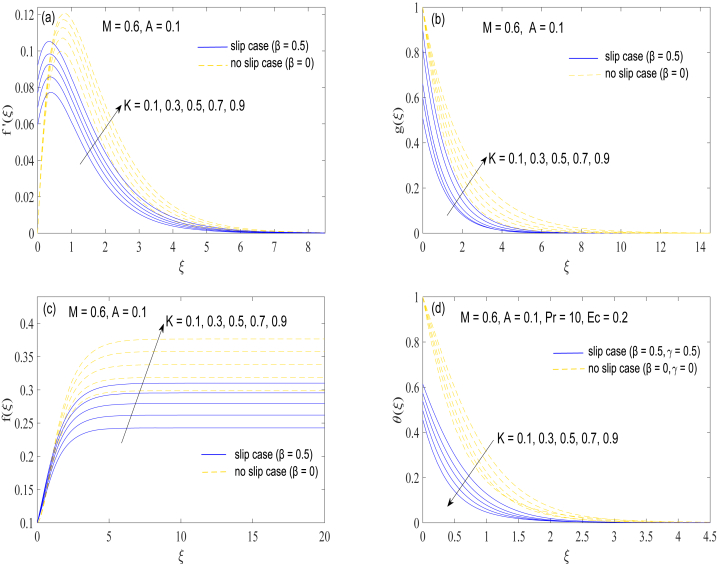
(a)-2(d): Changes in velocity components ($f^{\prime }\left(\xi \right),g\left(\xi \right)\;and\;f\left(\xi \right))$ and temperature θ(ξ) with the variation in elasticity parameter K.

Fig. 2(a)-2(d) display the computed velocity profiles $f,f^{\prime },g$ and temperature θ against ξ (the similarity variable) for changing values of K, the elasticity parameter. The elasticity parameter K is a measure of normal stress differences and its value is generally taken in range 0≤K≤1, in accordance with various previously published reports [26]. Similar to the conclusion of ref. [36], radial flow activated by the disk revolution becomes faster for rising values of K. More radial movement balances with more axial motion towards the disk (Fig. 2(c)). In addition, elasticity effects greatly assist the circumferential velocity component (Fig. 2(b)). Heat penetration depth is much shortened as parameter K enhances (Fig. 2(d)). It is an indirect effect brought about by the enhancement in axial movement (Fig. 2(c)), which improves the volume flow rate Q that leads to a decline in heat conduction process, as demonstrated through the plots of Fig. 2(d).

**Fig. 3:**
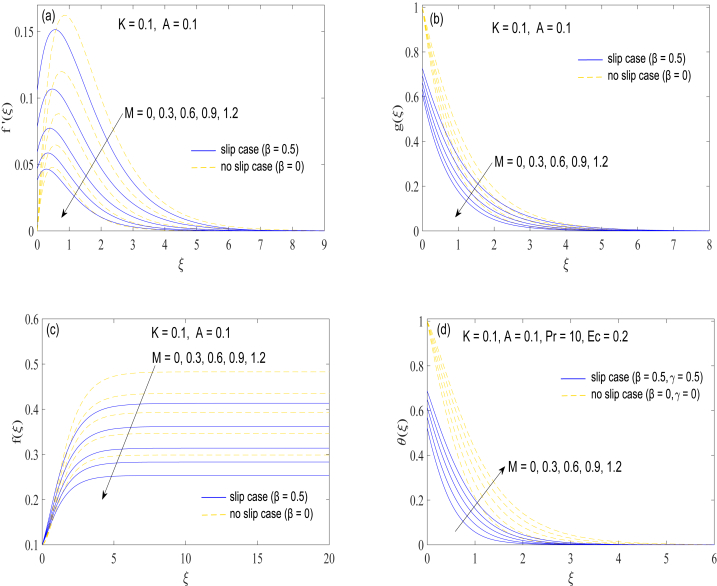
(a)-3(d): Changes in velocity components ($f^{\prime }\left(\xi \right),g\left(\xi \right)\;and\;f\left(\xi \right))$ and temperature θ(ξ) with the variation in magnetic interaction parameter M.

The behaviors of magnetic force on the flow and thermal fields are next presented in Fig. 3(a)-3(d). The intensity of the fluid and magnetic field's interaction is quantified through a magnetic interaction parameter M. The parameter M is typically taken to be modest, which suggests that the magnetic field's impact on the fluid is relatively weak in comparison to other forces acting on it. Naturally, the transverse magnetic field slows down the axial movement (Fig. 3(c)), which is balanced by a slower radial flow (Fig. 3(a)). Magnetic field affects the heat transport phenomena in two ways. Firstly, the weakened axial motion declines the volumetric flow rate thereby favoring the growth of thermal boundary layer. Secondly, Joule heating process improves when M enlarges, leading to the production of more heat. Temperature distribution is therefore expanded because of increase in M.

**Fig. 4:**
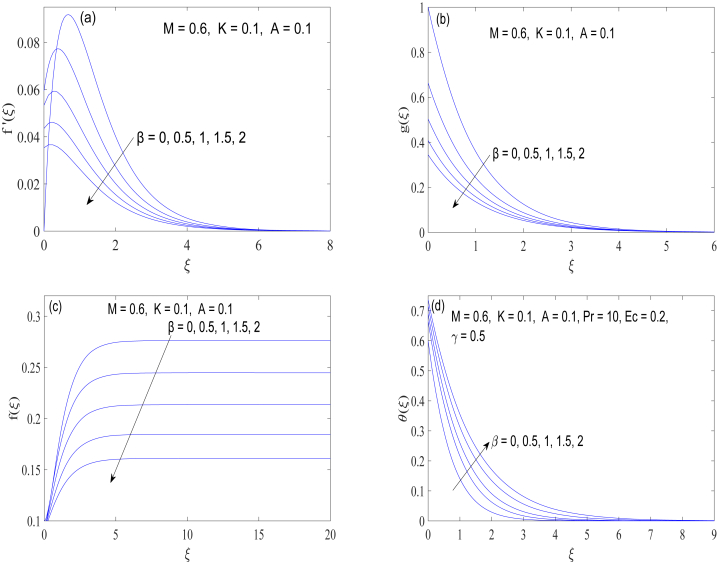
(a)-4(d): Changes in velocity components ($f^{\prime }\left(\xi \right),g\left(\xi \right)\;and\;f\left(\xi \right))$ and temperature θ(ξ) with the variation in velocity slip parameter β.

Behaviors of boundary slip on the flow and heat phenomena are next demonstrated in Fig. 4(a)-4(d). Whenever there is a relative motion between a fluid and the revolving disk, slip boundary condition is applied. In other words, there is a non-zero difference between the velocity of the fluid and velocity of the surface at the microscopic level. Slip effect is expected to become pronounced in the flow situations involving smooth surfaces. Slip coefficient $\lambda_{1}$ is a measure of characteristic distance over which fluid molecules adjust their velocity to match that of rotating disk surface. Slip parameter β therefore measures the strength of slip effect and it can take any value in the range 0<β<∞. The higher the slip coefficient, the greater the strength of the slip effect. The influence of slip mechanism on the streamwise momentum diffusion is remarkable. All velocity components are considerably declined as the wall slip coefficient enlarges. Physically speaking, revolution of the disk is only partially supplied to the fluid next to the surface, and as a result, boundary layer is markedly thinned upon inclusion of the slip mechanism. Fluid temperature raises substantially when the velocity slip feature is involved in the analysis (Fig. 4(d)).

**Fig. 5:**
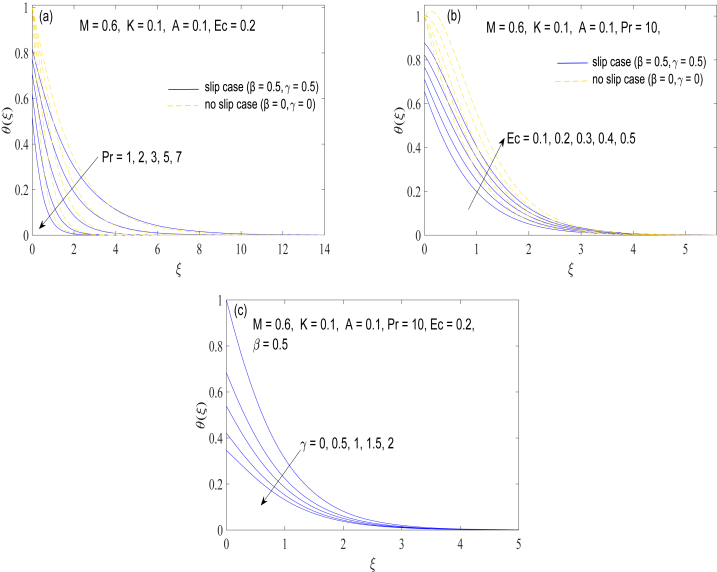
Profile of temperature θ(ξ) at different choices of (a) Prandtl number (b) Eckert number and (c) thermal slip parameter.

Fig. 5(a)-5(c) encompass the variation in temperature distribution θ(ξ) with ξ under different controlling parameters. The Prandtl number gives the relative importance of momentum diffusion (viscosity) over thermal diffusion (thermal conductivity) in a fluid. Non-Newtonian fluids including motor oils and granular liquids typically exhibit lower thermal conductivity and higher viscosity, leading to a higher Prandtl number (Pr≥7) being used to represent them. Naturally, contribution of heat conduction process reduces as Prandtl number grows. This is because of the inverse relationship between Pr and thermal diffusivity α. On the other hand, the consideration of frictional heating appreciably thickens the thermal penetration depth as illustrated in Fig. 5(b). The role of thermal slip is next presented in Fig. 5(c), which illustrates that heat transportation declines because of the thermal slip effects. Physically, the thermal slip effect causes the thin boundary layer near to the surface to get thinner. This thinned boundary layer improves heat transfer rate between the fluid and the solid surface.

**Fig. 6:**
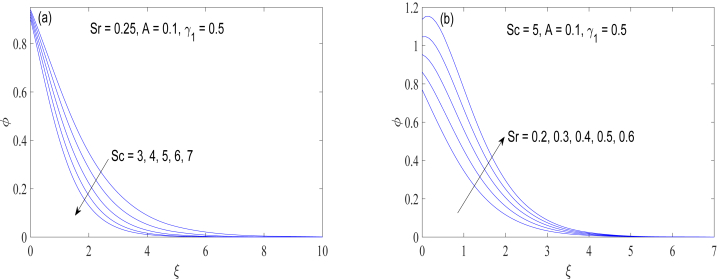
(a) & 6(b): Variation of concentration field φ(ξ) with different values of Schmidt number Sc and thermophoresis parameter Sr.

Graphs of concentration field φ by changing the Schmidt number Sc and the thermophoresis parameter Sr are computed in Fig. 6(a) and (b) respectively. Schmidt number Sc is an analog of Prandtl number for mass transfer. That is, it compares the thickness of concentration layer with that of momentum boundary layer. As Sc increases, curves of φ are moved closer to the wall revealing that concentration layer thickness is inversely proportional to Sc. Thermophoresis refers to the movement of suspended particles in response to the temperature gradient. Such movement occurs due to the differences in velocities of the fluid molecules and the suspended particles. For increasing Sr− values, mass diffusion becomes stronger owing to higher thermophoretic force, as illustrated in Fig. 6(b). Note that the parameters M,Ec and Sr represent the strengths of Lorentz force, viscous dissipation and thermophoresis effect respectively. The higher the values of these parameters, the greater their relative influence on the boundary layer. The values of M,Ec and Sr are taken in range 0 ≤M,Ec, Sr ≤∞.

**Fig. 7:**
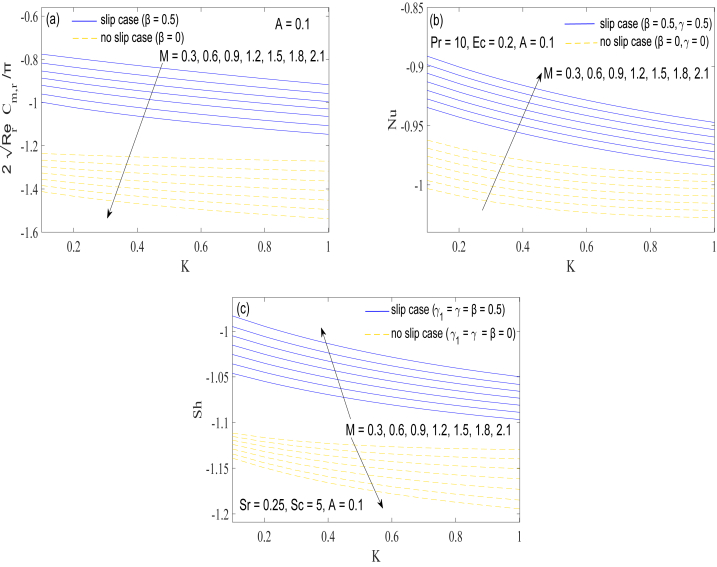
(a), 7(b) & 7(c): Effect of magnetic interaction parameter M and elasticity parameter K on the resisting torque, Nusselt number Nu and Sherwood number Sh**.**

Variation in the magnitude of torque required on the disk by changing elasticity parameter can be examined through Fig. 7(a). Intriguingly, almost a linear rise in resisting torque with parameter K is observed at all employed choices of M. Magnetic force also contributes to the growth in tangential stress. Nusselt number curves are computed against parameter K by varying the intensity of magnetic field. Heat transfer rate escalates sharply for rising values of normal stress differences or elasticity effects. However, presence of magnetic force has a deteriorating effect on the heat transfer rate, as elucidated from Fig. 7(b). Graphs of Sherwood number Sh are generated versus the elasticity parameter K by varying M in Fig. 7(c). Lorentz forces interact with fluid motion when a magnetic force is present in a fluid flow. These forces operate as a barrier to fluid flow, interrupting fluid velocity and hindering fluid particle mixing. As a result, the fluid's convective heat transmission is reduced. Magnetic force affects the mass transfer differently in no-slip and slip boundary conditions. However, contribution of elasticity on heat and mass transfer rates is qualitatively similar.

Table 3 demonstrates that wall slip declines the resisting torque and deteriorates heat transfer rate at the wall to a considerable extent. It is also noticed that disk requires more torque to maintain its rotation in a viscoelastic fluid, when compared with the usual Newtonian fluid case. This outcome complies with the observation reported in Ref. [36] for the no-slip case. Elasticity parameter raises the magnitude of Nusselt number, measuring heat transfer rate from the disk. Table 3 reveales the following information. The resisting torque experiences around 21% increase as the elasticity parameter K grows from 0 (representing Newtonian cases) to 1 (representing non-Newtonian cases), when the other parameters were kept fixed. Moreover, the resisting torque rises to about 40% as the magnetic interaction parameter M increases from 0 to 1.2 with other parameters fixed. It is observed further that the Nusselt number (Nu) undergoes an approximate 13% increase as the elasticity parameter K rises from 0 to 1 for a specific set of other parameters. However, the value of (Nu) experiences an approximate 37% decrease when the magnetic interaction parameter M increases from 0 to 1.2.

**Table 3:** Effects of elasticity parameter K, magnetic interaction parameter *M,* velocity slip parameter β and thermal slip parameter γ on resisting torque and Nusselt number when A=0.1,Pr=10andEc=0.2.

K	M	β	γ	$\frac{2}{\pi }{Re_{r}}^{\frac{1}{2}}C_{m,r}$	Nu
0.1	0.6	0.5	0.5	−0.68071	0.63272
0.3				−0.71312	0.65388
0.5				−0.74695	0.67033
0.7				−0.78107	0.68343
0.9				−0.81476	0.69410
0.1	0			−0.55996	0.80789
	0.3			−0.62070	0.71584
	0.6			−0.68071	0.63272
	0.9			−0.73509	0.56324
	1.2			−0.78282	0.50737
	0.6	0		−0.99510	0.48114
		0.5		−0.68071	0.63272
		1		−0.50222	0.63644
		1.5		−0.39792	0.62030
		2		−0.32994	0.60158
		0.5	0	−0.68071	1.00694
			0.5	–	0.63272
			1	–	0.46128
			1.5	–	0.36295
			2	–	0.29917

Table 4 shows how mass diffusivity and thermophoresis effects alter the mass transfer rate of the disk lying in viscoelastic fluid. When Schmidt number is higher, mass diffusion is weaker compared to momentum diffusion and concentration profile is thinner (as also seen in Fig. 6(a)). The thinner the concentration profile, the higher the transfer rate. Furthermore, mass transfer rate is markedly declined when the thermophoretic force is introduced.

**Table 4:** Effect of Schmidt number Sc, Soret number Sr and concentration slip parameter $\gamma_{1}$ on Sherwood number $-\varphi^{\prime }\left(0\right)$.

Sc	Sr	$\gamma_{1}$	Sh
3	0.25	0.5	0.24605
4			0.27142
5			0.28971
6			0.30355
7			0.31439
5	0.1		0.53394
	0.2		0.45253
	0.3		0.37112
	0.4		0.28971
	0.5		0.20829
	0.25	0	0.44460
		0.3	0.33661
		0.6	0.27084
		0.9	0.22656
		1.2	0.19473

## 5 Major outcomes of the research

This study looked at the slip flow of a second-grade fluid over a permeable revolving disk with combined heat and mass transfer. Computations for the self-similarity equations are presented for wide range of embedded parameters. Salient observations of present research are appended below.

• As in the no-slip case, the fluid flow accelerates whenever elasticity effects are included. On the other hand, the temperature is considerably reduced due to the increase in the elasticity parameter.
• Due to the presence of transverse magnetic field, the velocity (all components) is clearly reduced. Physically, the magnetic field induces a resistive force called the Lorentz force, whose strength increases for increasing M-values.
• For increasing values of M, the contribution of ohmic heating to heat generation improves, leading to a much broader temperature profile.
• The velocities (all components) are clearly suppressed under the action of wall slip mechanism. The implication of temperature/concentration slip trend is such that heat/mass penetration is substantially reduced due to the increase in the thermal/concentration slip coefficient.
• A noticeable increase in driving torque is witnessed when the elasticity parameter enhances. Slip action of the surface also adds to the driving torque.
• Thermal/concentration slip parameter is beneficial in improving cooling/mass transfer rate of the surface/disk.
• In future, Present study may be explored using other viscoelastic fluid models such as Jeffrey fluid model, Maxwell fluid model and others. Also, an interesting Bodewadt flow situation can also be studied using the second-grade fluid model.

## Author contribution statement

Haleema Sadia, M. Mustafa: Conceived and designed the analysis; Analyzed and interpreted the data; Contributed analysis tools or data; Wrote the paper.

## Data availability statement

No data was used for the research described in the article.

## Declaration of competing interest

The authors declare that they have no known competing financial interests or personal relationships that could have appeared to influence the work reported in this paper.

## References

[1] Carreau P.J. (1972). Rheological equations from molecular network theories. Trans. Soc. Rheol..

[2] Ostwald W. (1929). de Waele-Ostwald equation. Kolloid Z..

[3] R. S. Rivlin and J. L. Ericksen, Stress-Deformation Relations for Isotropic Materials. In: G. I. Barenblatt and D. D. Joseph (eds) Collected Papers of R.S. Rivlin, Springer, New York, NY. 10.1007/978-1-4612-2416-7_61.

[4] Kaloni P.N., Siddiqui A.M. (1983). The flow of a second grade fluid. Int. J. Eng. Sci..

[5] Rajagopal K.R., Na T.Y., Gupta A.S. (1984). Flow of a viscoelastic fluid over a stretching sheet. Rheol. Acta.

[6] Pontrelli G. (1995). Flow of a fluid of second grade over a stretching sheet. Int. J. Non Lin. Mech..

[7] Hsu C.H., Hu S.Y., Kung K.Y., Kuo C.C., Chang C.C. (2013). A study on the flow patterns of a second grade viscoe-lastic fluid past a cavity in a horizontal channel. J. Mech..

[8] Mustafa M., Abbasbandy S., Hina S., Hayat T. (2014). Numerical investigation on mixed convective peristaltic flow of fourth grade fluid with Dufour and Soret effects. J. Taiwan Inst. Chem. Eng..

[9] Ramesh K., Devakar M. (2017). Effect of heat transfer on the peristaltic transport of a MHD second grade fluid through a porous medium in an inclined asymmetric channel. Chin. J. Phys..

[10] Hina S., Yasin M. (2018). Slip effects on peristaltic flow of magnetohydrodynamics second grade fluid through a flexible channel with heat/mass transfer. J. Therm. Sci. Eng. Appl..

[11] Roy N.C., Pop I. (2020). Flow and heat transfer of a second-grade hybrid nanofluid over a permeable stretching/shrinking sheet. Eur. Phys. J. Plus.

[12] Abbas N., Tumreen M., Shatanawi W., Qasim M., Shatnawi T.A.M. (2023). Thermodynamic properties of second grade nanofluid flow with radiation and chemical reaction over slendering stretching sheet. Alex. Eng. J..

[13] Adeniyan A., Mabood F., Okoya S. (2021). Effect of heat radiating and generating second grade mixed convection flow over a vertical slender cylinder with variable physical properties. Int. Commun. Heat Mass Tran..

[14] Raees A., Farooq U., Hussain M., Khan W.A., Farooq F.B. (2021). Non-similar mixed convection analysis for magnetic flow of second-grade nanofluid over a vertically stretching sheet. Commun. Theor. Phys..

[15] Hayat T., Ahmad N., Sajid M., Asghar S. (2007). On the MHD flow of a second grade fluid in a porous channel. Comput. Math. Appl..

[16] Majeed A., Javed T., Ghaffari A. (2016). Numerical investigation on flow of second grade fluid due to stretching cylinder with Soret and Dufour effects. J. Mol. Liq..

[17] Shojaei A., Amiri A.J., Ardahaie S.S., Hosseinzadeh K., Ganji D.D. (2019). Hydrothermal analysis of Non-Newtonian second grade fluid flow on radiative stretching cylinder with Soret and Dufour effects. Case Stud. Therm. Eng..

[18] Von-Kármán T. (1921). Uber laminare and turbulente Reibung. Zeitschrift fur Angew Math. & Mech..

[19] Lok Y.Y., Merkin J.H., Pop I. (2018). Axisymmetric rotational stagnation-point flow impinging on a permeable stretching/shrinking rotating disk. Eur. J. Mech. B Fluid.

[20] Turkyilmazoglu M. (2018). Fluid flow and heat transfer over a rotating and vertically moving disk. Phys. Fluids.

[21] Mushtaq A., Mustafa M. (2017). Computations for nanofluid flow near a stretchable rotating disk with axial magnetic field and convective conditions. Res. Phys..

[22] Mandal S. (2021). G.C. Shit, Entropy analysis on unsteady MHD biviscosity nanofluid flow with convective heat transfer in a permeable radiative stretchable rotating disk. Chin. J. Phys..

[23] Hussain M., Rasool M., Mahmood A. (2022). Radiative flow of viscous nano-fluid over permeable stretched swirling disk with generalized slip. Sci. Rep..

[24] Waini I., Ishak A., Pop I. (2022). Multiple solutions of the unsteady hybrid nanofluid flow over a rotating disk with stability analysis. Eur. J. Mech. B Fluid.

[25] Anuar N.S., Bachok N., Pop I. (2021). Radiative hybrid nanofluid flow past a rotating permeable stretching/shrinking sheet. Int. J. Numer. Methods Heat Fluid Flow.

[26] Elliott L. (1971). Elastico-viscous flow near a rotating disk. Phys. Fluids.

[27] Ariel P.D. (1997). Computation of flow of a second grade fluid near a rotating disk. Int. J. Eng. Sci..

[28] Andersson H.I., de Korte E. (2002). MHD flow of a power-law fluid over a rotating disk. Eur. J. Mech. B Fluid.

[29] Ahmadpour A., Sadeghy K. (2013). Swirling flow of Bingham fluids above a rotating disk: an exact solution. J. Non-Newtonian Fluid Mech..

[30] Guha A., Sengupta S. (2016). Analysis of von Karman swirling flow on a rotating disk in Bingham fluids. Phys. Fluids.

[31] Sahoo B., Poncet S., Labropulu F. (2015). Suction/injection effects on the swirling flow of a Reiner-Rivlin fluid near a rough surface. J. Fluids.

[32] Tabassum M., Mustafa M. (2018). A numerical treatment for partial slip flow and heat transfer of non-Newtonian Reiner-Rivlin fluid due to rotating disk. Int. J. Heat Mass Tran..

[33] Rashid M.U., Mustafa M. (2021). A study of heat transfer and entropy generation in von Kármán flow of Reiner-Rivlin fluid due to a stretchable disk. Ain Shams Engg. Journal.

[34] Rafiq T., Mustafa M., Khan J.A. (2022). Rotationally symmetric flow of Reiner-Rivlin fluid over a heated porous wall using numerical approach. Proc. iMeche Part C: J. Mech. Eng. Sci..

[35] Jafeer M.B., Mustafa M. (2021). A study of elastico-viscous fluid flow by a revolving disk with heat dissipation effects using HAM based package BVPh 2.0. Sci. Rep..

[36] Jafeer M.B., Mustafa M. (2022). A novel formulation and analysis for heat transfer in von Kármán flow involving viscoelastic fluid: OHAM solutions. J. Therm. Analys. & Calor..

[37] Hina S., Mustafa M., Abbas Z., Kayani S.M. (2022). Numerical simulations for heat transfer in peristalsis of Bingham fluid utilizing partial slip conditions. Waves Random Complex Media.

[38] Showkat I., Mushtaq A., Mustafa M. (2023). Numerical exploration of buoyancy inspired flow of pseudoplastic fluid along a vertical cylinder with viscous dissipation effects. Alex. Eng. J..

